# Blast in Context: The Neuropsychological and Neurocognitive Effects of Long-Term Occupational Exposure to Repeated Low-Level Explosives on Canadian Armed Forces' Breaching Instructors and Range Staff

**DOI:** 10.3389/fneur.2020.588531

**Published:** 2020-12-03

**Authors:** Oshin Vartanian, Catherine Tenn, Shawn G. Rhind, Ann Nakashima, Alex P. Di Battista, Lauren E. Sergio, Diana J. Gorbet, Douglas D. Fraser, Angela Colantonio, Kristen King, Quan Lam, Doug Saunders, Rakesh Jetly

**Affiliations:** ^1^Defence Research and Development Canada, Toronto Research Centre, Toronto, ON, Canada; ^2^Department of Psychology, University of Toronto, Toronto, ON, Canada; ^3^Defence Research and Development Canada, Suffield Research Centre, Medicine Hat, AB, Canada; ^4^Faculty of Kinesiology and Physical Education, University of Toronto, Toronto, ON, Canada; ^5^School of Kinesiology and Health Science, York University, Toronto, ON, Canada; ^6^Department of Clinical Neurological Sciences, Western University, London, ON, Canada; ^7^Rehabilitation Sciences Institute, Toronto, ON, Canada; ^8^Canadian Forces Health Services, Ottawa, ON, Canada

**Keywords:** TBI, blast, concussion, military personnel, cognitive motor integration

## Abstract

Currently, there is strong interest within the military to better understand the effects of long-term occupational exposure to repeated low-level blast on health and performance. To gain traction on the chronic sequelae of blast, we focused on *breaching*—a tactical technique for gaining entry into closed/blocked spaces by placing explosives and maintaining a calculated safe distance from the detonation. Using a cross-sectional design, we compared the neuropsychological and neurocognitive profiles of breaching instructors and range staff to sex- and age-matched Canadian Armed Forces (CAF) controls. Univariate tests demonstrated that breaching was associated with greater post-concussive symptoms (*Rivermead Post Concussion Symptoms Questionnaire*) and lower levels of energy (*RAND SF-36*). In addition, breaching instructors and range staff were slower on a test that requires moving and thinking simultaneously (i.e., cognitive-motor integration). Next, using a multivariate approach, we explored the impact of other possible sources of injury, including concussion and prior war-zone deployment on the same outcomes. Concussion history was associated with higher post-concussive scores and musculoskeletal problems, whereas deployment was associated with higher post-concussive scores, but lower energy and greater PTSD symptomatology (using PCL-5). Our results indicate that although breaching, concussion, and deployment were similarly correlated with greater post-concussive symptoms, concussion history appears to be uniquely associated with altered musculoskeletal function, whereas deployment history appears to be uniquely associated with lower energy and risk of PTSD. We argue that the broader injury context must, therefore, be considered when studying the impact of repetitive low-level explosives on health and performance in military members.

## Introduction

Recent military engagements in Iraq and Afghanistan have been associated with significant rates of blast-induced traumatic brain injury (TBI). For example, according to statistics from the Department of Defense (DoD), 14% of TBI cases encountered in Operation Enduring Freedom (OEF) and/or Operation Iraqi Freedom (OIF) were due to blast exposure (1). Indeed, based on data compiled by the Department of Veterans Affairs, nearly three-quarters of all combat-related injuries over the period 2005–2009 were due to explosions (2). Importantly, 10–15% of TBI cases from those theaters of war continue to report persistent post-concussive symptoms following the resolution of the initial symptoms (3), indicating that TBI represents an enduring public health concern for our service members and Veterans.

Accordingly, neurological impairments, including mild TBI, are increasingly recognized as an occupational health and performance concern within the Canadian Armed Forces (CAF) and the Canadian Special Operations Forces Command (CANSOFCOM) (4, 5). However, isolating the effects of low-level blast in theater has proven difficult because of the tremendous heterogeneity that exists in the nature of explosions and their effects on individuals in combat settings (6). For example, it is recognized that blast-induced TBI can result from multiple factors, such as direct exposure to the explosive wave, projectiles that penetrate the skin, structural collapse or displacement of the body, and/or indirect effects such as thermal exposure—referred to as primary, secondary, tertiary and quaternary effects of blast exposure, respectively (7). In this sense, it is difficult to tease apart and measure the effects of primary blast exposure from other accompanying factors in combat settings.

**Figure 1:**
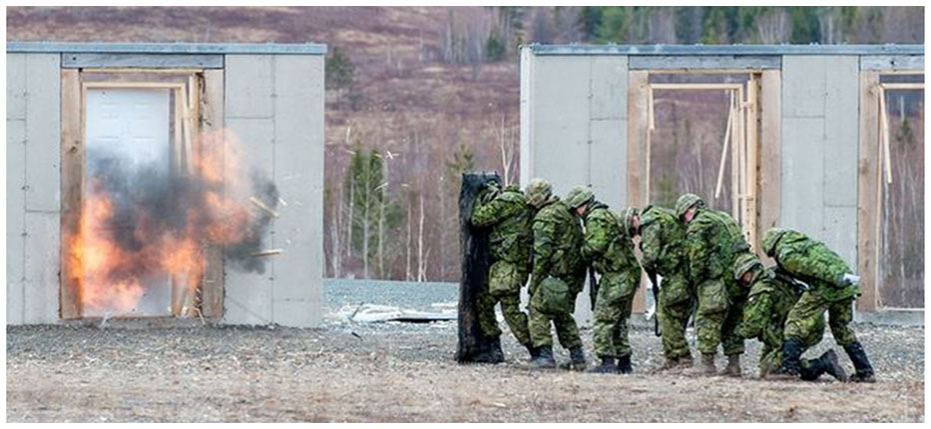
Canadian Forces School of Military Engineering personnel participating in breaching exercises. Photo courtesy of Haley Voutour (5th Canadian Division Support Group).

Because the conditions that characterize blast exposure in operational settings complicate one's ability to study the effects of primary blast *in situ*, researchers have explored surrogate settings wherein the effects of exposure to blast can be assessed in an operationally realistic, yet scientifically more controlled manner. One such context involves explosive breaching, which is a tactical technique used to gain entry into a closed or blocked space using explosives [see (8)]. The procedure involves the placement of explosives and the maintenance of a calculated distance away from the source during detonation (Figure 1). Exposure levels during breacher training can vary depending on the charge weight, reflective surfaces in the environment, the geometry of the structures involved, and location of the exposed individual relative to the explosion. Nevertheless, breacher training is regulated by guidelines in order to limit hazardous exposure to blast overpressures in trainees, breaching instructors and range staff. For example, according to the Canadian Army's tactical breaching manual, breachers should not be exposed to blast overpressures that exceed a threshold of 3 pounds per square inch (psi) (9). However, a recent preliminary examination using blast gauges mounted on Canadian Forces School of Military Engineering (CFSME) instructors and range staff during breacher training revealed that ~12% of blast events exceeded 3 psi (10). This naturalistic observation suggested that despite adherence to guidelines that govern breacher training, it is nevertheless possible for individuals to be exposed to potentially hazardous levels of blast, with possible downstream effects on health and performance.

Although the precise nature of the relationship between long-term exposure to repetitive low-level blast and human health remains unknown (11–13), data suggest that long-term exposure to blast events can have adverse effects on the nervous system (14, 15), and can be associated with alterations in cerebral metabolism, diffuse white matter disruption, chronic neuroinflammation (16, 17), or perturbations to circulating levels of neurological injury biomarkers (8, 18–21). Indeed, a major theme in the literature revolves around whether blast injuries represent a different mechanism of injury than acceleration–deceleration injuries, by virtue of their physical dynamics. This idea is plausible, given the effect that explosives can have on both air-filled organs and/or organs surrounded by fluid-filled cavities within the body (22, 23), and remains an important area of study [see Belding et al. (24)].

Critically, despite lack of clarity regarding the underlying mechanism of injury, self-reports of breachers reveal concussion-like symptoms including headaches, sleep disturbances, and memory impairments that can interfere with daily activity (19, 25). In addition, and particularly relevant to the present purposes, there is reason to believe that the impairments do not arise in relation to acute exposure, but rather accumulate as a function of repetitive, cumulative exposure to low-level blast over the course of one's career. For example, the number and severity of symptoms reported by breachers increases with their history of chronic blast exposure (19). In addition, it has been shown that it is breaching instructors who oversee training regularly, rather than students who partake in as few as a single training exercise, that exhibit impairments in various memory tasks and alterations in brain function. Specifically, there was greater activation in the prefrontal cortex when performing a working memory task in instructors but not students following participation in a 2-week breacher course, compared to baseline (25). Because the impairment and associated neural alterations were specific to instructors, it appears that they emerge in response to repeated occupational exposure to low-level blast in the course of one's career, rather than acutely following exposure to isolated blast events [see also (15)].

### Present Study

The objective of the present study was to investigate the impact of long-term occupational exposure to repeated low-level blast on health and performance in CAF members. Toward that end, we administered a battery of neuropsychological and neurocognitive measures to breaching instructors and range staff from CFSME, and compared their scores and performance to a group of sex- and age-matched CAF controls with no occupational experience with breaching. The selection of measures was largely informed by the literature on blast-induced TBI and concussion in sports [see (26)]. Regarding the latter, we administered a measure derived from the sports concussion literature that has not been used to study the impact of blast on performance in the past. Specifically, the *Brain Dysfunction Indicator* (BrDI) is a device that measures performance on a task that requires moving and thinking at the same time—known as ‘cognitive-motor integration'. BrDI has been shown to be sensitive to movement control impairments in individuals with a history of concussion. These impairments are apparent in several aspects of the task such as movement reaction time, completion time, accuracy, and precision, and are detected despite the individuals showing no impairments in other tests that are currently available for assessing concussion recovery. Its ability to sensitively detect performance impairment in cognitive-motor integration has been shown for elite and competitive athletes—both adult (27, 28) and youth (29, 30). In addition, there is recent evidence to suggest an association between cognitive-motor performance and white matter integrity (31)—a structural neural index that might be affected by repetitive exposure to low-level blast. BrDI was, therefore, included in our task battery because of its sensitivity to detect concussion in athletes, and also to complement the remainder of our neurocognitive tasks, all of which measured various aspects of cognition and perception exclusively, rather than integrated.

Aside from our focal interest on the impact of repetitive exposure to low-level blast on health and performance, we were also cognizant of the fact that the same outcome measures could be affected by other sources of (head) injury—in particular concussion and deployment to a war zone. There were two reasons for this conjecture; first, as described above, some of our metrics for measuring the impact of blast in military personnel were informed by the sports literature on concussion. As such, one might expect that a history of concussion will influence those outcomes. Second, because war-zone deployment can be associated with a variety of health hazards, it is possible that any measure that reflects impairments in health might also be affected by one's deployment history. More broadly, we understand that blast effects on health and performance occur within a larger professional and personal context, and that it is important to probe those pathways as well, in order to obtain a more holistic picture of the impact of blast on military personnel exposed to explosives.

We hypothesized that compared to sex- and age-matched CAF controls, breaching instructors and range staff would exhibit impairments measured by tests of neuropsychological and neurocognitive function. As part of our battery we also included a measure of clinical posttraumatic stress disorder (PTSD) symptomatology [PCL-5, (32)]. This measure was included because previous studies with service members and veterans have shown that there might be comorbidity between blast-induced TBI and PTSD and/or depression, among other clinical symptoms [e.g., (33–36), see also (37)]. In the present study we did not expect to see any differences between the two groups on PCL-5, since we had no a priori reason to believe that exposure to breacher training *per se* is an emotionally traumatic experience. However, regardless of breaching, we did suspect that deployment to a war zone would be associated with elevated scores on the PCL-5.

## Method

### Participants

The study protocol was approved by the Human Research Ethics Committee of Defence Research and Development Canada. Potential participants were recruited via an electronic recruitment poster that was circulated among CFSME staff (for breaching instructors and range staff) and at Denison Armory (for controls). If interested in participating in the study, members were asked to email the PI. The participants were breaching instructors and range staff (*n* = 19) from CFSME, and sex- and age-matched CAF controls with no occupational experience as breachers (*n* = 19). Their demographics and service history appear in Table 1.

**Table 1:** Demographics and service history.

**Variables**	**Breachers/range staff (*n* = 19)**	**CAF controls (*n* = 19)**	**Mean difference (95% CI)**	**Bootstrap ratio**	***P***
Age (years)	33 (27-38)	32 (27.5–35.5)	0.8 (−4.4–5.7)	3.2	0.742
Sex–(*n*, % male)	17 (89.5)	17 (89.5)	0 (−21.1–21.1)	0	0.790
Military service (years)	11.3 (9–14.5)	5 (1.5–10.2)	6.4 (3.2–10.3)	3.7	**<0.001**
Exposure to explosives (years)	10 (7.5–12)	0 (0–0)	10.4 (8–13.1)	8.0	**<0.001**
Breaching (years)	7 (4.5–10)	0 (0–0)	7.1 (5.2–9.3)	7.2	**<0.001**
Combat deployment	11 (64.7)	0 (0)	68.2 (47.4–89.5)	6.3	**<0.001**
**Status**				
Regular Force	9 (47.4)	8 (42.1)	5.8 (−21.1–31.6)	0.4	0.546
Reservist	10 (52.6)	11 (57.9)	5.8 (−21.1–31.6)	0.4	0.546
**Rank**				
Junior NCM	5 (26.3)	13 (68.4)	−42.4 (−68.4 to −10.5)	2.8	**0.004**
Senior NCM	12 (63.2)	0 (0)	63 (42–84.5)	5.4	**<0.001**
Junior Officer	2 (10.5)	6 (31.6)	−20.6 (−47.4–10.5)	1.4	0.098
**Education**				
High School	6 (31.6)	4 (21.1)	10.6 (−21.1–42.1)	0.7	0.378
College	6 (31.6)	4 (21.1)	10.5 (−15.8–36.8)	0.8	0.310
Undergraduate	5 (26.3)	10 (52.6)	−26 (−52.6–5.3)	1.7	0.056
Graduate	1 (0.5)	1 (0.5)	0.4 (−15.8–15.8)	0	0.658
None	1 (0.5)	0	5.2 (0–15.8)	1	0.312

*Continuous/integer data presented as the median and interquartile range–med (iqr); categorical data presented as the frequency and percent = n (%). CAF, Canadian Armed Forces; NCM, Non-commissioned member. Significance corrected at a false discovery rate of p = 0.05 (**bold p-values)**, derived from bootstrapped mean difference testing. For categorical variables, the mean difference was evaluated on the percent of individuals categorized to each outcome (see section Methods)*.

There has been no prior quantification of the amount of blast that instructors and range staff are exposed to in the course of their careers at CFSME, although some parameters can be used to contextualize the problem space. CFSME administers between 8 and 20 breaching courses per year. In turn, each course includes 1–2 days of breaching on the range (see Figure 1). Typically, instructors (also sometimes referred to as Assistant Range Safety Officers [ARSOs]) and range staff form a “cell” that administers the courses together for a period of 1–3 years. In that period, and unless there is a scheduling conflict, members of each cell will be at the range together. Nevertheless, the specific amount of blast overpressure that members within the same cell are exposed to can vary, depending on various factors such as one's geographic position, functional role, and the geometry of the space (which can impact wave reflection and re-convergence, etc.), among other factors. The instructors are integrated into the breaching stack for both wall and door breaches, although their position within the stack can vary depending on the condition. According to current breaching guidelines, the maximum number of exposures an instructor can be exposed to is limited to six blast events per day. After this limit has been reached, the instructors are rotated out of the stack and serve other functions on the range further away from the source of the blast in order to limit additional exposure. In turn, range staff who are not instructors but fulfill other roles on the range (e.g., Officer in Charge of the Range, Range Safety Officer, Ammunition NCO, etc.) are typically further away from the source of the detonations, and therefore receive relatively less exposure than instructors. However, they may be exposed to more than six blasts events per day. In summary, the magnitude and number of blast events that breaching instructors and range staff are exposed to can vary, the quantification of which will be an important step for improving our understanding of the etiology of blast-induced TBI.

### Materials and Procedure

All data were collected in a single session for each participant. CFSME breachers and range staff were tested at Canadian Forces Base Gagetown (CFB Gagetown). Sex- and age-matched CAF controls were tested at DRDC (Toronto Research Center). The measures included the neuropsychological and neurocognitive tasks discussed here, as well as a suite of physiological indices (i.e., blood biomarkers, hearing, vestibular function, and postural tremor). Findings in relation to physiological measures will be discussed in a separate manuscript.

The *Background Health Questionnaire* included six questions to assess history of prior head injury (Table 2). The participants completed a battery of neuropsychological measures. The *RAND SF-36 Health Survey* (38) has 36 items aggregated into 8 health-related scales, where a score of 100 indicates optimal functioning in that health category. The *Short Musculoskeletal Function Questionnaire* **[**SMFQ; (39)] generates scores on two indices: The *Dysfunction Index* (DI) assesses the participant's perceptions of his or her functional musculoskeletal performance, whereas the *Bother Index* (BI) assesses how much the participant is bothered by musculoskeletal problems. The participants completed a modified version of the *Rivermead Post Concussion Symptoms Questionnaire* [RPQ; (40)]. Specifically, rather than asking participants to compare themselves to a time prior to the accident, for each symptom they were asked to indicate whether they had experienced it as a function of injury to the head. Next, symptomatic criteria for PTSD were assessed using the 20-item *Post-Traumatic Checklist* (PCL-5), according to the Diagnostic and Statistical Manual of Mental Disorders [DSM-5, (32)].

**Table 2:** History of prior head injury.

**Variables**	**Breachers/range staff (*n* = 19)**	**CAF controls (*n* = 19)**	**Mean difference (95% CI)**	**Bootstrap ratio**	***P***
Concussion	8 (44.4)	5 (26.3)	21 (−5.3–47.4)	1.5	0.088
Physical impact to head	9 (47.4)	11 (57.9)	−10.4 (−36.8–15.8)	0.7	0.402
MVA	14 (73.7)	9 (47.4)	2.6 (−5.3–52.6)	1.7	0.066
Fallen as child	8 (42.1)	6 (31.6)	10.4 (−10.5–31.6)	1	0.206
Physical fight	13 (68.4)	15 (78.9)	−10.6 (−31.6–15.8)	0.9	0.258
Blast exposure	19 (100)	2 (10.5)	89.2 (73.7–100)	12.5	**<0.001**

*CAF, Canadian Armed Forces; MVA, motor vehicle accident. Data presented as the frequency and (%). Significance corrected at a false discovery rate of p = 0.05 (bold p-values), derived from bootstrapped mean difference testing evaluated on the percent of individuals categorized to each outcome (see section Method)*.

**Figure 2:**
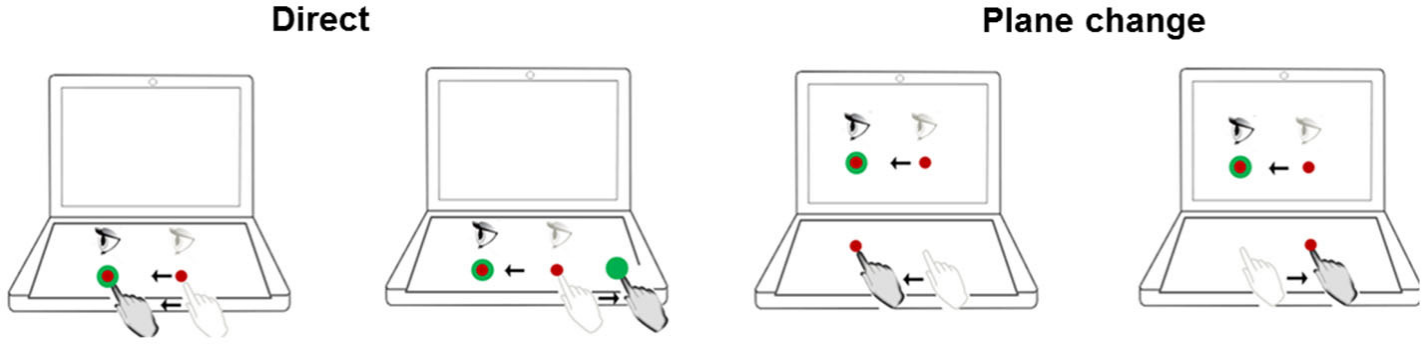
Schematic of experimental conditions for BrDI. BrDI, Brain Dysfunction Indicator. Visual stimuli were presented either directly on the computer tablet (same plane; Direct) or on the attached monitor (plane change). Light gray eye and hand indicate the start position. The dark gray eye and hand symbols depict the movement from start position toward the target. The target was presented in one of four locations (right, left, up or down).

Neurocognitive function was assessed using the Cognitive Test Software (41). This involved the computerized administration of four measures in sequence: (1) *Delayed matching-to-sample (dMTS)*: This test assessed short-term visual (iconic) memory and pattern recognition (42). (2) *Four-choice reaction time task (4-choice RT task)*: This test assessed the ability to respond rapidly and accurately to simple visual stimuli presented on a computer screen (43). (3) *n-back*: This is a test of working memory performance, and requires the maintenance and updating of dynamic rehearsal sets (44). In the present study, *n* had a range of 1-3. (4) *Stroop*: This is a test of executive functions, specifically inhibition (45). Finally, the participants completed BrDI (Figure 2). For that task participants were seated at a desk with a touch-sensitive computer tablet connected to an external monitor. While wearing a touch-screen glove on their dominant hand, participants were instructed to place their finger on a central spot on the horizontally placed computer tablet and move the cursor as accurately and quickly as possible across the screen into the target. In two of the four conditions, participants viewed the targets directly on the tablet while sliding their finger in the same or opposite direction to move the cursor toward the target. In the other two conditions, participants viewed the targets and cursor on an external monitor in the vertical upright position while moving their finger in the same or opposite direction. Each participant performed 5 trials in each of 4 randomly presented conditions. Finally, all participants completed *Cognistat* (46–48) which is a measure used to assess cognitive function in five distinct ability areas, including their subcomponents (language, spatial-constructional skills, memory, calculations, and reasoning and judgment). The test requires 15–20 min for completion. The test format was paper-and-pencil, administered individually by one of two experimenters who were trained to criterion in advance.

### Statistical Analysis

Prior to statistical analysis, all variables were checked for deviations from normality through testing the skewness and kurtosis for each variable against a random gaussian noise model (1,000 iterations). Skewness in the breacher/range staff group ranged from 0.2 (*p* = 0.630) to −2.6 (*p* < 0.001), whereas kurtosis ranged from 2.4 (*p* = 0.997) to 15.5 (*p* < 0.001). In CAF controls, skewness ranged from 0 (*p* = 0.834) to 3 (*p* < 0.001), and kurtosis ranged from 3 (*p* = 0.871) to 23 (*p* < 0.001). Hence, before statistical testing, variables exhibiting moderate normality deviations were transformed by winsorization (10%), whereas variables that severely deviated from normality were rank transformed.

Univariate, between-group comparisons for continuous/interval variables (demographic, psychological and cognitive measures) were conducted using a bootstrapped mean difference test (1,000 resamples), run in a repeated-measures framework to account for CAF subject matching. Briefly, a distribution of mean difference scores for each variable was created to identify the average and 95% confidence interval of the difference between groups; percentile *p*-values were obtained by computing the fraction of bootstrapped coefficient values not enclosing zero effect in a two-tailed framework, which were then corrected at a false discovery rate (FDR) of 0.05. Standardized effect sizes were defined in terms of bootstrap ratios (BSR) which were calculated by dividing the bootstrapped mean of the differences by the standard error of the mean for each comparison. For categorical variables, the mean difference test was evaluated on the percent of individuals categorized to each outcome. For example, given a binary variable with two possible outcomes (0 or 1), the difference in the percent of individuals with outcome 1 was calculated between groups.

To compare psychological and cognitive test profiles (1) between breachers/range staff and CAF controls, (2) between personnel with vs. without a history of concussion, and (3) between personnel who were deployed vs. never deployed to a war zone, a partial least squares discriminant analysis (PLSDA) test was employed. PLSDA is a classification algorithm that seeks to maximize covariance between a set of predictor variables (cognitive and psychological test scores) and a single binary response variable (breacher/non-breacher; concussion/no concussion; war zone/no war zone). By creating latent variables comprised of individual variable weights, the PSLDA is optimized to handle collinearity, and is well-posed for a low ratio of subjects-to-variables. The PLSDA was run in a bootstrapped framework (1,000 iterations), followed by the generation of effect sizes as bootstrap ratios (BSR: mean/standard error) and percentile *p*-values, corrected at an FDR of 0.05. Model performance was evaluated via predication accuracy (Accur) and posterior probability (PProb) estimates, procured in a leave-two-out resampling framework. Accur was evaluated by assigning each subject to the outcome group with the most similar PLS score, and then quantitating the percent of correctly classified subjects. PProb was derived via the calculated likelihood of the PLS model in identifying the correct outcome conditioned on observed subject scores under a Gaussian noise model. Prior to PLSDA analyses, the potential confounding influence of age was adjusted for by partial regression on all affected variables, and any variables with near-zero variance were removed prior to analysis. All data were analyzed and graphed using R (RStudio, version 1.2.1335, Boston, United States).

## Results

### Demographics and Service History

Demographic and service history variables in breachers/range staff and CAF controls can be seen in Table 1. Both groups were predominantly male (89.5%), with breachers/range staff reporting significantly more years of military service (BSR = 3.7, *p* < 0.001), years of breaching (BSR = 7.2, *p* < 0.001) and a history of combat deployment (BSR = 6.39, *p* < 0.001) than CAF controls. Breachers/range staff were comprised of a higher proportion of Senior NCM ranked personnel compared to CAF controls (BSR = 5.4, *p* < 0.001), whereas CAF controls were comprised of a higher proportion of Junior NCM ranked individuals (BSR = 2.8, *p* = 0.004).

### History of Prior Head Trauma

Head trauma history in breachers/range staff and CAF controls are displayed in Table 2. There were no differences in prior head trauma history between the two groups with the exception of blast exposure which, as expected, was prevalent in 100% of breachers/range staff but only in 10.2% of CAF controls (BSR = 12.5, *p* < 0.001).

### Neuropsychological and Neurocognitive Measures

Breachers/range staff and CAF controls' neuropsychological and neurocognitive scores can be seen in Table 3. Breachers/range staff scored significantly lower on the Energy subscale of SF-36 (BSR = 2.2, *p* = 0.022) compared to CAF controls. Rivermead scores were analyzed using two methods. First, responses to the initial three items of the questionnaire (headache, feelings of dizziness, nausea/vomiting) generated RPQ-3 that captures *early* post-concussive symptoms (i.e., symptoms that tend to present themselves closer to the time of injury), whereas responses to the next thirteen items (e.g., sleep disturbance) generated RPQ-13 that captures *late* post-concussive symptoms (i.e., symptoms that tend to present themselves later following the injury) (49). Second, we sorted the items into cognitive, emotional, and somatic symptoms. Items in each category were summed, omitting scores of “1” (40). Following Verfaellie et al. (50), we divided the total score for each category by its number of items. Breachers/range staff scored significantly higher on both the RPQ3 (BSR = 3.8, *p* < 0.001) and RPQ13 (BSR = 4.0, *p* < 0.001) compared to CAF controls; scores were also higher for Rivermead's somatic (BSR = 3.6, *p* = 0.004), cognitive (BSR = 2.9, *p* = 0.004) and emotional (BSR = 3.4, *p* < 0.001) test components in breachers/range staff compared to CAF controls.

**Table 3:** Neurocognitive and neuropsychological measures.

**Variables**	**Breachers/range staff (*n* = 19)**	**CAF controls (*n* = 19)**	**Bootstrap ratio**	**Fractional *P***
**Neuropsychological Measures**				
**RAND SF-36**				
General health	75 (67.5–80)	80 (65–92.5)	0.7	0.478
Physical functioning	95 (92.5–100)	100 (95–100)	1.6	0.120
Emotional well-being	80 (66–88)	84 (68–88)	0.4	0.676
Social functioning	100 (87.5–100)	100 (7–100)	0.3	0.786
Pain	90 (80–90)	90 (85–100)	1.5	0.126
Energy	50 (37.5–67.5)	65 (60–80)	2.2	0.022
Role limitations (physical health)	100 (100–100)	100 (100–100)	0	0.952
Role limitations (emotional problems)	100 (100–100)	100 (66.7–100)	1.2	0.224
**SMFA**				
Function index	40 (37–45)	34 (34–41)	2.4	0.016
Bother index	15 (13–16.5)	12 (12–16)	1.8	0.072
**Rivermead**				
RPQ3	2 (1–5.5)	0 (0–2)	3.8	**<0.001**
RPQ13	7.0 (1.5–15.5)	0 (0–2.5)	4.0	**<0.001**
Somatic	0.6 (0.1–1.1)	0 (0–0.1)	3.6	**0.004**
Cognitive	0 (0–1.3)	0 (0–0)	2.9	**0.004**
Emotional	0 (0–0.9)	0 (0–0)	3.4	**<0.001**
PCL-5	7 (0.5–10.5)	0 (0–6)	1.9	0.068
**Cognistat**–**frequency (%)**				
Consciousness	19 (100)	19 (100)	–	–
Attention	16 (84.2)	16 (84.2)	0.1	0.742
Memory	19 (100)	16 (84.2)	1.9	**<0.001**
Comprehension	19 (100)	16 (84.2)	1.9	**<0.001**
Repetition	16 (84.2)	16 (84.2)	0	0.810
Naming	13 (68.4)	11 (57.9)	0.8	0.306
Constructional ability	17 (89.5)	16 (84.2)	0.6	0.362
Calculations	14 (73.7)	16 (84.2)	0.8	0.346
Similarities	18 (94.7)	15 (78.9)	1.4	0.126
Judgement	13 (68.4)	16 (84.2)	1.2	0.150
**Neurocognitive measures**				
4-choice RT task (ms)	451 (406–519.5)	450 (425.5–517)	0.7	0.538
**n-back (d')**				
1-back	4.7 (3.8–4.7)	4 (3.4–4.7)	1.2	0.218
2-back	2.5 (2.1–3.1)	2.8 (2.2–3.5)	0.7	0.490
3-back	1.2 (0.9–1.6)	1.3 (1.2–1.7)	1.1	0.254
dMTS_(% correct)	68 (56–78)	72 (66–82)	1.6	0.094
Stroop (ms)	48 (16.2–80.2)	49 (38–62.5)	0.1	0.918
**BrDI (msec)**				
Same plane veridical	365.8 (335.6–409.3)	343.2 (282.6–358.9)	2.7	**0.004**
Same plane reversed	511.9 (461.0–560.6)	479.6 (397.6–528.3)	1.3	0.182
Differential plane veridical	386.3 (355.0–452.5)	340.2 (304.7–369.4)	3.0	**0.002**
Different plane reversed	528.9 (460.8–645.1)	477.3 (409.6–514.4)	2.2	0.034

*Interval and continuous data presented as the median and interquartile range–med (iqr); categorical data presented as the frequency and percent–n (%). BrDI, Brain Dysfunction Indicator; RT, reaction time; ms, milliseconds; dMTS, delayed matching-to-sample task. For n-back and dMTS the numbers indicate accuracys (percentage); for Stroop the numbers indicate reaction time (seconds). Significance corrected at a false discovery rate (FDR) of p = 0.05 (bold p-values), derived from bootstrapped mean difference testing. For categorical variables, the mean difference was evaluated on the percent of individuals categorized to each outcome (see section Methods)*.

**Figure 3:**
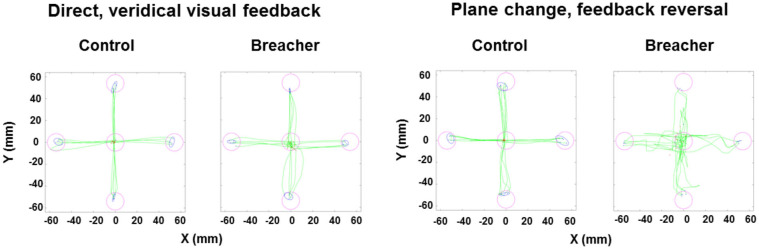
Examples of hand movement trajectories for BrDI. BrDI, Brain Dysfunction Indicator. Examples of hand movement trajectories (green) in the Direct condition (i.e., movements in target direction and in the same plane) and in the Plane change feedback reversal condition (i.e., movements in the opposite direction of the presented target and in a different plane) from one control participant (left side of each condition panel) and from one breaching instructor or range staff (right side of each condition panel). Magenta circles represent the location of targets. Red dots indicate the starting position of the finger for each trial. Blue ellipses represent the 95% confidence intervals for the final endpoint positions of movements (blue dots).

**Figure 4:**
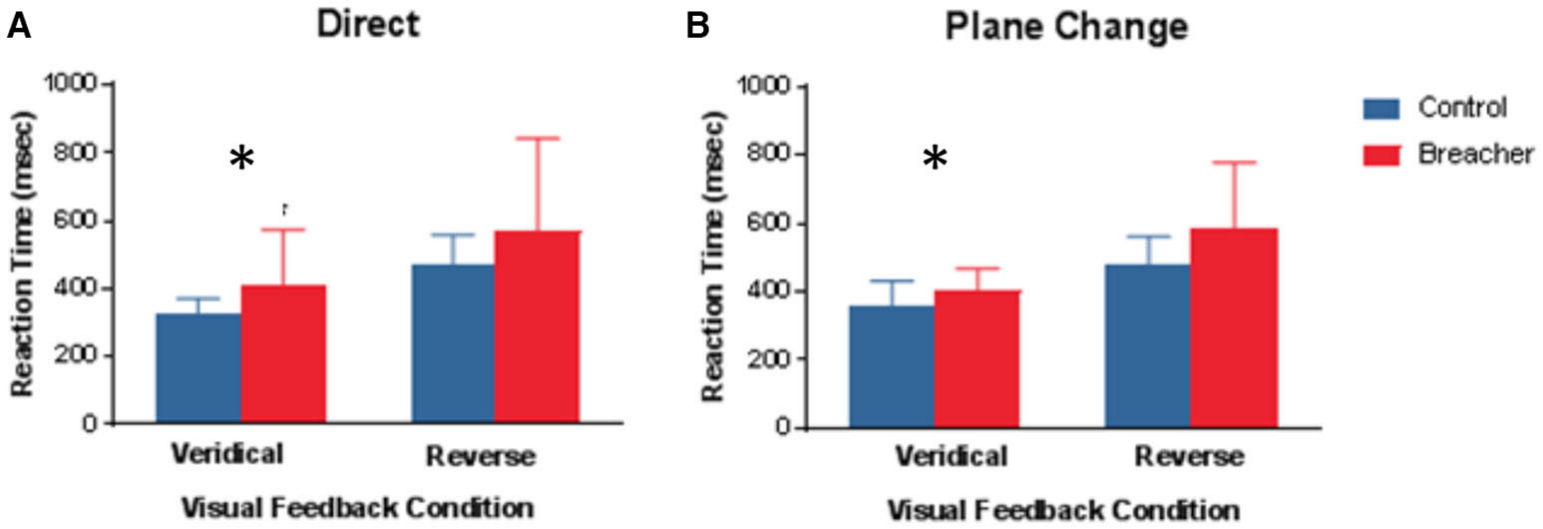
Mean Reaction time under 4 different conditions for BrDI. BrDI, Brain Dysfunction Indicator. **(A)** Same plane with veridical (left panel) or reversed (right panel) visual feedback to move the cursor into the target. **(B)** Different plane with veridical (left panel) or reversed (right panel). Error bars represent ± SD. Significant difference between breaching instructors and range staff vs. controls (see text) *p < 0.01.

Examples of hand movement trajectories associated with performance on BrDI are illustrated in Figure 3. The dependent variable for this task consisted of RT associated with the four conditions. BrDI direct plane veridical (BSR = 2.7, *p* = 0.004) and differential plane veridical (BSR = 3.0, *p* = 0.002) times were significantly higher in breachers/range staff compared to CAF controls (Figure 4).

Tasks on the neurocognitive task battery were scored as follows: For dMTS, the dependent variable was accuracy (i.e., percentage correct out of 25 trials). For the 4-choice RT task, the dependent variable was the RT associated with correct responses. For Stroop, the dependent variable was the difference in RT for correctly identifying the color of incongruent word trials (e.g., the word RED appearing in blue) vs. RT for correctly identifying the color of congruent word trials (e.g., the word RED appearing in red). For the n-back, the dependent variable was *d*' (i.e., sensitivity) (51). Higher *d*' values reflect greater sensitivity, whereas a *d*' nearing zero reflects chance performance. None of these measures appeared sensitive to the effects of blast. However, on Cognistat, CAF controls displayed significantly higher failure rates on tests of memory and comprehension compared to breachers/range staff (BSR = 1.9, *p* < 0.001 for both tests).

**Figure 5:**
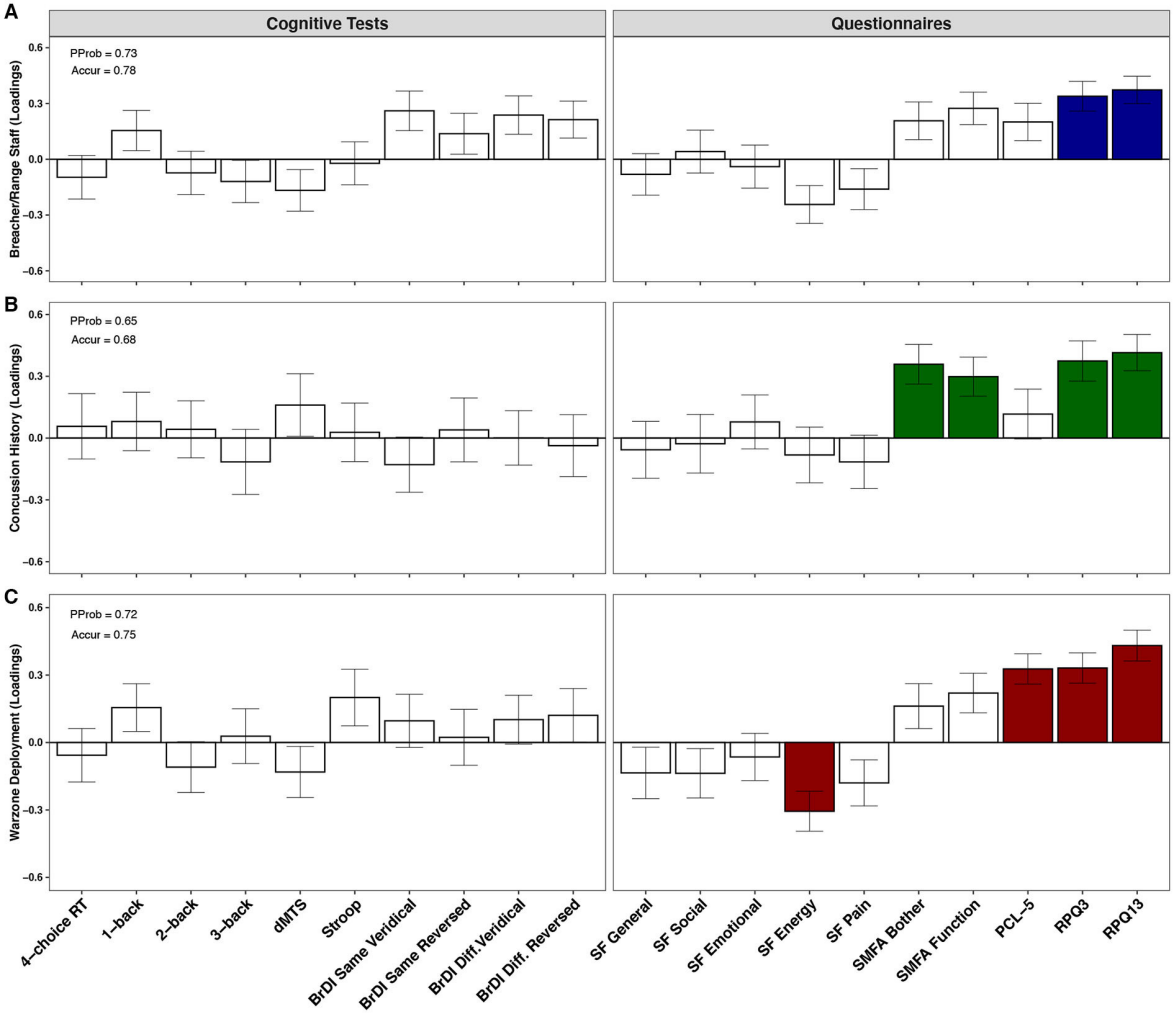
Psychological and cognitive test profiles of military personnel. PProb, posterior probability; Accur, accuracy; RT, reaction time; dMTS, delayed matching-to-sample task; BrDI, brain dysfunction indicator; SMFA, selective functional movement assessment; PCL-5, Posttraumatic stress disorder (PTSD) checklist for diagnostic and statistician's manual (DSM-5); RPQ, Rivermead post-concussion symptoms questionnaire. Plots show the contributions of psychological and cognitive measures toward class separation in **(A)** breachers/range staff (*n* = 19) vs. CAF controls (*n* = 19), **(B)** personnel with (*n* = 13) vs. without (*n* = 24) a history of concussion, and **(C)** personnel deployed to a war zone (*n* = 11) vs. never deployed (*n* = 25), by partial least squares discriminant analysis (PLSDA). Bars represent biomarker loadings and the standard error derived from bootstrapped resampling (1,000 samples). Colored bars = significant at a false discovery rate (FDR) < 0.05.

PLSDA plots displaying psychological and cognitive test profiles in three separate classification analyses are shown in Figure 5. All analyses were adjusted for the effects of age. RPQ3 (BSR = 4.5, *p* < 0.001) and RPQ13 (BSR = 5.1, *p* < 0.001) scores contributed significantly to class separation between breachers/range staff (*n* = 19) and CAF controls (*n* = 19), with higher scores found in the former; the PProb of the model was 0.73, and Accur was 0.78 (Figure 5A). When all participants were taken into consideration (i.e., breachers/range staff and CAF controls combined), those with a history of concussion (*n* = 13) displayed higher scores on RPQ3 (BSR = 3.6, *p* = 0.006) and RPQ13 (BSR = 4.5, *p* = 0.002), as well as higher scores on SMFA's Bother Index (BSR = 3.8, *p* < 0.001) and Dysfunction Index (BSR = 3.2, *p* < 0.001), compared to those with no history of concussion (*n* = 24); model PProb was 0.65, and Accur was 0.68 (Figure 5B). Finally, compared to those who had never deployed to a war zone (*n* = 25), deployment to a war zone (*n* = 11) was associated with lower scores in the SF-36 Energy subscale (BSR = 3.3, *p* = 0.006), as well as higher scores in the PCL-5 (BSR = 4.8, *p* < 0.001), the RPQ3 (BSR = 4.7, *p* < 0.001) and RPQ13 (6.2, *p* < 0.001); model PProb was 0.72 and Accur was 0.75 (Figure 5C).

## Discussion

Our study was conducted to test the hypothesis that compared to sex- and age-matched CAF controls, breachers and range staff would exhibit functional impairments measured on standardized neuropsychological and neurocognitive tests. Indeed, univariate analyses demonstrated that compared to CAF controls, breachers and range staff reported significantly greater post-concussive symptoms (*Rivermead Post Concussion Symptoms Questionnaire*), as well as lower levels of energy (*RAND SF-36 Health Survey*). These results suggest that repetitive exposure to low-level blast is associated with impairments in health and function as measured by self-report neuropsychological measures.

None of our standard tests of neurocognitive function that measure short-term visual memory, choice reaction time, or executive functions proved sensitive to the effects of repetitive exposure to low-level blast. In contrast, breachers and range staff exhibited longer RT in two conditions on BrDI task. This novel finding suggests that cognitive-motor integration might actually be an ability that is affected adversely by blast exposure, and that BrDI represents a useful method for detecting the impact of blast exposure over and above what can be obtained using standard neurocognitive tasks alone. Accordingly, there has been considerable fundamental research on the underlying brain activity during cognitive-motor integration, and how this is distinct from brain activity associated with thinking alone or moving alone. These studies used tasks that are laboratory versions of what is tested with BrDI, and support the scientific concept underlying the unique nature of this approach (52–57). Although no previous human studies in military blast have specifically examined this functional measure, a considerable body of work suggests that there is a unique additive value to including tasks that measure cognitive-motor integration in studies of the effects of blast exposure (12, 37).

Importantly, despite impairment in cognitive-motor integration, breaching instructors and range staff are nevertheless able to perform demanding jobs. Hurtubise et al. (28) have noticed a similar pattern in concussed elite athletes. Specifically, they reported noticeable behavioral deficits in elite vs. non-elite concussed athletes, despite elite athletic performance in the former group. It was proposed that high-level athletes possess a superior fronto-parietal network connectivity due to their higher level of training, and are, therefore, able to compensate for the mild brain injury. Similarly, it is possible that CAF breachers and range staff have also built-up superior fronto-parietal networks following years of military training, and can thereby perform at high levels, occupationally. This interpretation is consistent with their scores on Cognistat, where they performed better on the subcomponents of Memory and Comprehension than CAF controls. These findings suggest that there might indeed be components of cognitive function that could be enhanced by the occupational demands of breaching, the mechanisms for which require further study.

An important aspect of our approach in this study was to examine the impact of blast within the larger occupational context of military service and injury. Our multivariate approach demonstrated that the disturbances found in breachers and range staff do not appear to be unique to breaching, as they were also observed, to a similar extent, in military personnel with a history of concussion as well as those who have been deployed to a war zone. Specifically, it appears that post-concussive symptoms are associated with all three conditions: breaching, concussions, and war-zone deployment (Figure 5). However, within a non-matched, age-adjusted model, reported concussion history appears to uniquely alter SMFA scores, whereas war-zone deployment appears to uniquely alter perceived energy and risk of PTSD. In the case of PCL-5 measures, individuals with multiple deployments showed consistently higher scores (median value of 7), which corresponds with greater PTSD symptomatology (58). Although well-below the threshold for clinical diagnosis (i.e., PCL-5 score ≥ 33), this observation is consistent with a large body of research suggesting a link between multiple deployments, mTBI and increasing vulnerability to developing PTSD and other mental health problems (59, 60). These findings should prove useful as researchers work toward developing improved diagnostic tools for distinguishing between the effects of these three conditions that frequently overlap in this population exposed routinely to low-level blast (61, 62).

It is important to exercise caution in interpreting our findings. First, although our two groups were comparable in terms of demographic and past brain injury indicators, they differed on a number of factors that might have affected our findings, including greater number of years of service in the military, as well as a greater frequency of deployment to war. Second, because we employed a quasi-experimental cross-sectional design, it is not possible to draw any causal inference from our findings. However, we do hope that our findings will motivate longitudinal studies that are better suited for isolating the effects of long-term occupational exposure to repeated low-level blast in operators [see Kamimori et al. (21)]. Third, because the precise mechanism(s) underlying blast-related neurological injury remains unknown, additional work on that fundamental problem is necessary for gaining a better understanding of the injury pathway (12, 37). Fourth, our sample reflects an armed forces population that is mostly male, and as such the findings may not be entirely representative of females (63–66). Despite these limitations, our results suggest that long-term occupational exposure to repeated low-level blast is a phenomenon that requires further systematic study, and that outcomes associated with it might not be necessarily unique to the breaching environment.

## Data Availability Statement

The datasets presented in this article are not readily available because the data for this study were collected from members of the Canadian Armed Forces (CAF). Permission from the CAF's chain of command will be required to make the generated dataset available for public access. Requests to access the datasets should be directed to Oshin Vartanian, oshin.vartanian@drdc-rddc.gc.ca.

## Ethics Statement

The studies involving human participants were reviewed and approved by Defence Research and Development Canada Human Research Ethics Committee. The participants provided their written informed consent to participate in this study.

## Author Contributions

OV, CT, SR, AN, LS, DF, AC, and RJ designed the study. OV, CT, SR, AN, KK, QL, and DS collected the data. OV, AD, DG, and KK analyzed the data. All authors contributed to writing and editing the manuscript.

## Conflict of Interest

The authors declare that the research was conducted in the absence of any commercial or financial relationships that could be construed as a potential conflict of interest.
